# Cardiac Arrest Following Treatment With Diltiazem for Atrial Fibrillation With Rapid Ventricular Response

**DOI:** 10.7759/cureus.11678

**Published:** 2020-11-24

**Authors:** Woo Kyung Lee, Masra Shameem, Latha Ganti, Paul R Banerjee, John Shivdat

**Affiliations:** 1 Emergency Medicine, Mercer University School of Medicine, Macon, USA; 2 Emergency Medicine, University of Central Florida College of Medicine, Orlando, USA; 3 Emergency Medicine, Envision Physician Services, Plantation, USA

**Keywords:** atrial fibrillation, ventricular tachycardia, av block, cardiopulmonary resuscitation

## Abstract

We present the case of a 43-year-old man with a history of atrial fibrillation and poor medical compliance who presented to the emergency department with palpitations for three hours. Electrocardiogram (ECG) revealed atrial fibrillation with rapid ventricular response at 119 beats per minute. Following administration of diltiazem 10 mg IV, the patient became bradycardic with a rate of 30 beats per minute and complete atrioventricular node block. A subsequent ECG revealed asystole, and the patient became unresponsive. Chest compressions were administered, and the rhythm changed to ventricular tachycardia. There was spontaneous return of circulation without any further intervention. The patient eventually converted to sinus rhythm and was started on anticoagulation to prevent a thrombotic event. He was discharged the next day with apixaban and propafenone.

## Introduction

Atrial fibrillation (AF) is the most common type of cardiac arrhythmia worldwide [1]. It is characterized by disorganized and rapid electrical activity in the atria of the heart, which causes its impaired function. There are a multitude of factors that lead to the development of AF; however, underlying pathologies that alter the anatomy or electrical physiology of the atria account for most cases. These underlying risk factors include hypertension, valvular disease, coronary artery disease, congenital heart disease, excessive alcohol use, and advanced age. Patients may be asymptomatic and diagnosed during routine care, whereas symptomatic patients experience palpitations, chest pain, shortness of breath, dizziness, or fatigue.

The goal of treatment for AF is to prevent a thromboembolic event such as a stroke. This is achieved through anticoagulation with rate or rhythm control. For hemodynamically stable patients, pharmacological agents used to control the rapid ventricular response (RVR) include beta-blockers (BBs) and calcium channel blockers (CCBs), such as diltiazem. We present a case of cardiac arrest after diltiazem use for rate control in a patient with AF.

## Case presentation

A 43-year-old male presented with palpitations that started three hours prior to presentation. He had a similar episode three years ago at which time he was diagnosed with AF with RVR. During that episode, he was treated with diltiazem 10 mg and the rhythm converted to sinus rhythm. After that episode, he was taking metoprolol, but had stopped taking it about two years ago. He had not followed up with a cardiologist and had not had another episode in the prior two years.

The patient was alert and oriented but complained of palpitations associated with mild nausea. The patient had been generally healthy and did not take any medication. He was diagnosed with influenza A one week prior to presentation and finished a five-day course of oseltamivir. He denied illegal drug use and endorsed occasional coffee consumption. Physical examination was unremarkable except for an irregularly irregular rhythm and tachycardia. His laboratory evaluation was also unremarkable, including a complete blood count, chemistry panel, and thyroid-stimulating hormone level. 

**Figure 1:**
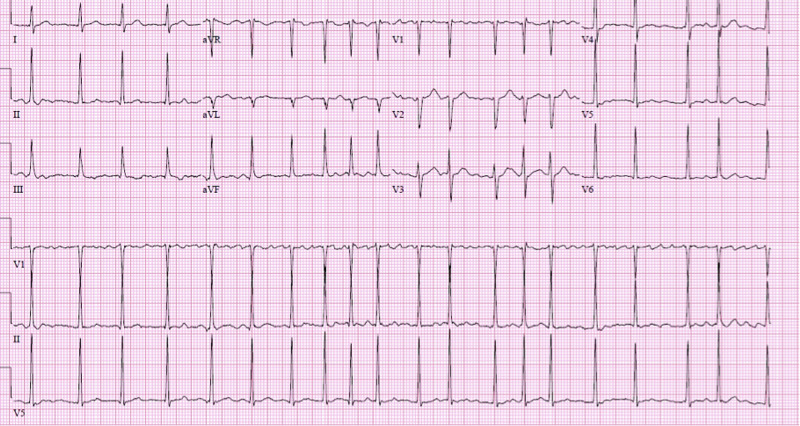
Electrocardiogram demonstrating atrial fibrillation with rapid ventricular response

Upon arrival to the ED, his vital signs were as follows: blood pressure of 124/89 mmHg, pulse of 119 beats per minute (bpm), respirations of 18 breaths per minute, and saturation of 98% on room air (SpO_2_). Electrocardiogram (ECG) revealed AF with RVR (Figure 1).

**Figure 2:**
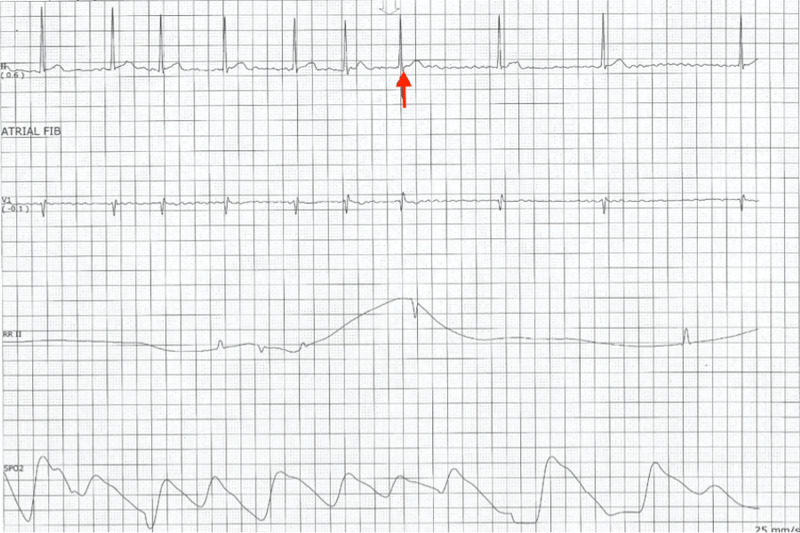
Electrocardiogram after diltiazem administration (red arrow)

Diltiazem 10 mg was ordered, and the patient was placed under telemonitoring. The second ECG was obtained, at which time vital signs were as follows: blood pressure of 103/69 mmHg, pulse of 80 bpm, SpO_2_ of 98% (Figure 2).

**Figure 3:**
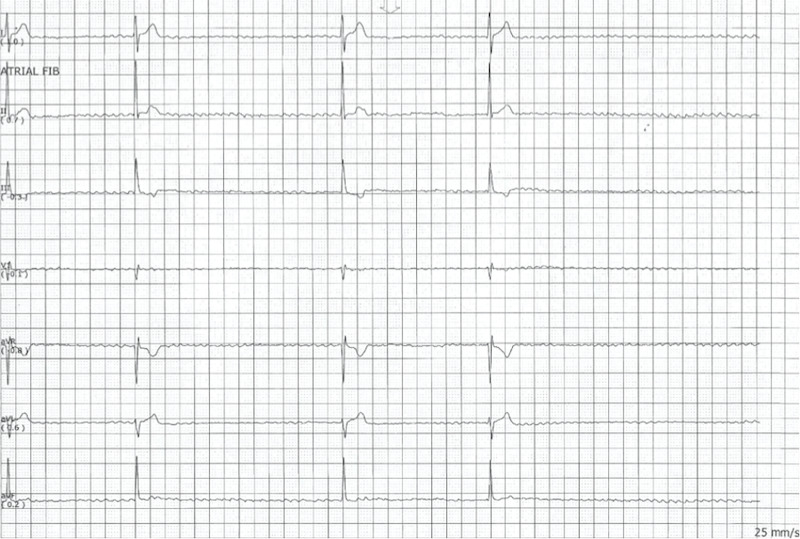
Electrocardiogram demonstrating asystole after diltiazem administration

The heart rate then decreased to less than 30 bpm, and the rhythm showed a complete atrioventricular (AV) block. The patient subsequently became asystolic and unresponsive (Figure 3).

**Figure 4:**
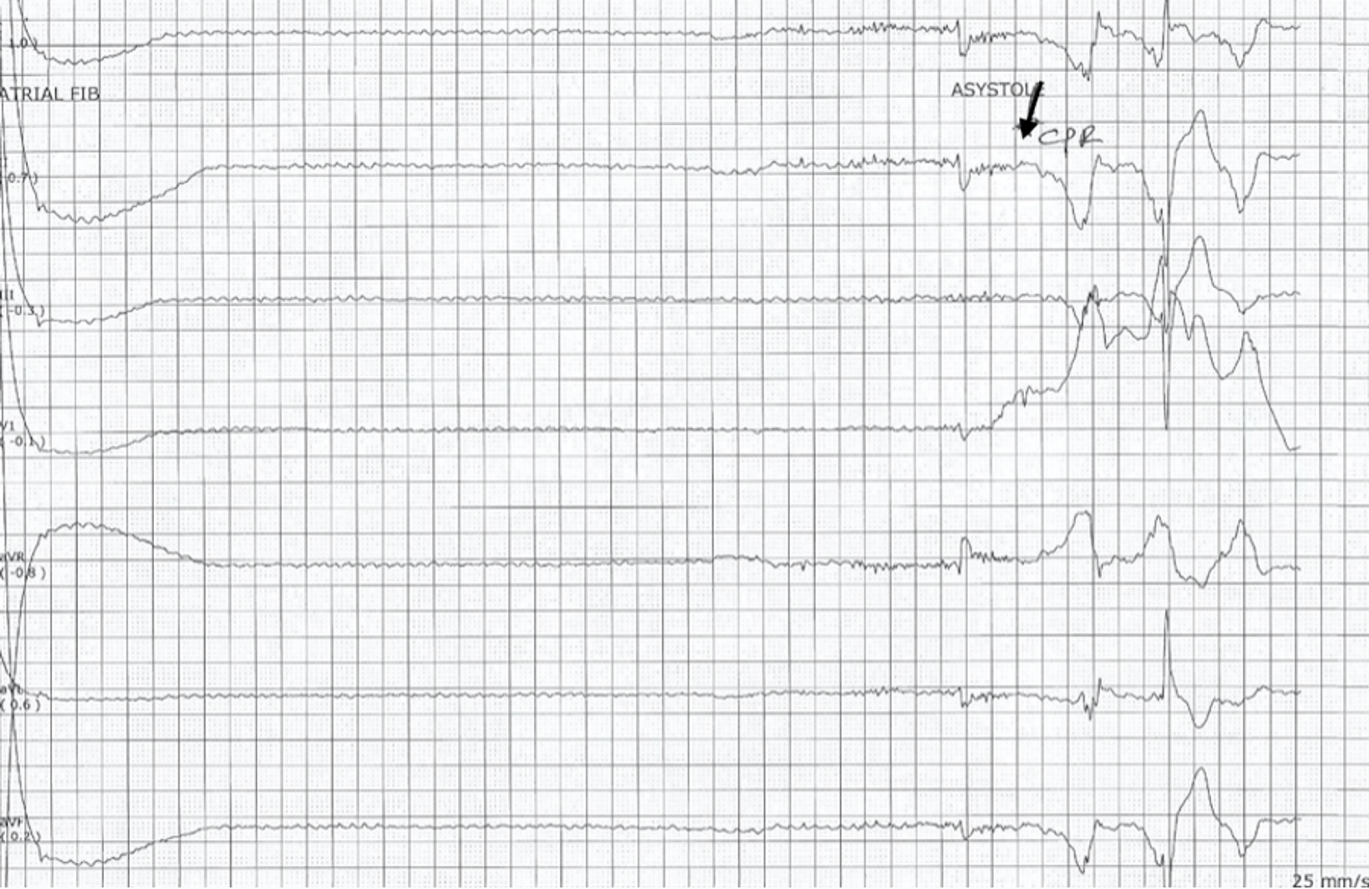
Electrocardiogram demonstrating rhythm change from asystole to ventricular tachycardia

Chest compressions were started, and rhythm subsequently changed from asystole to ventricular tachycardia (Figure 4).

**Figure 5:**
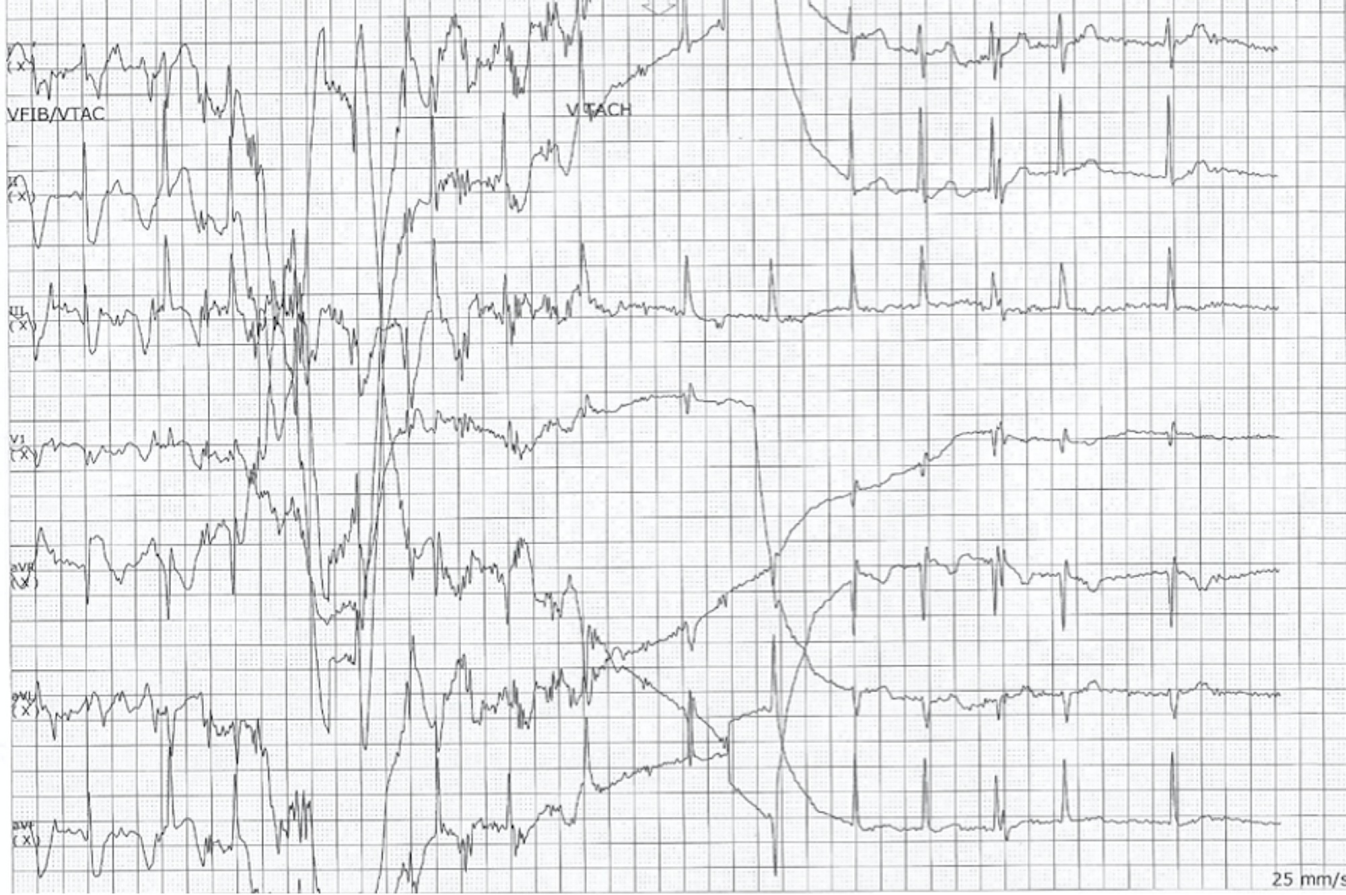
Electrocardiogram at the time the patient had return of spontaneous circulation

The patient had return of spontaneous circulation (ROSC) within a minute without interventions such as epinephrine or defibrillation (Figure 5).

**Figure 6:**
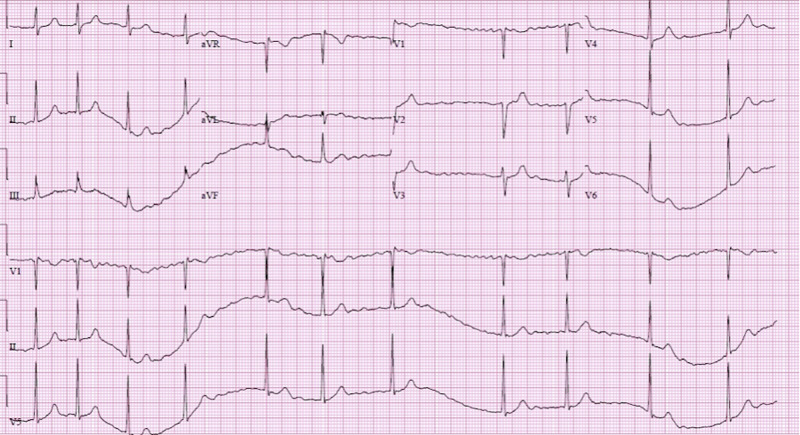
Electrocardiogram demonstrating atrial fibrillation with a normal rate

Vitals at that time showed blood pressure of 124/82 mmHg, heart rate of 99 bpm, and SpO_2_ of 99%. The patient then woke up and was alert and oriented without any neurologic deficits, but he was unable to remember what had happened. After the rhythm was stabilized, the ECG showed a heart rate of 67 bpm and AF (Figure 6).

Electrophysiology service was consulted, and the patient was admitted to the hospital for further evaluation. Two-dimensional echocardiography was performed which showed normal systolic function (50-60% ejection fraction), normal diastolic function, no valve abnormalities, and no pericardial effusion. The patient had a CHADS-VASc score of 0, but given that he was in AF for several hours, he would need anticoagulation for at least 30 days to reduce his risk of thromboembolism. The patient was thus discharged home the next day with apixaban 5 mg and propafenone 150 mg both twice daily.

## Discussion

This case report describes a patient with a recurrence of AF and subsequent cardiac arrest following rate control therapy. AF can be categorized as paroxysmal, persistent, long persistent, and permanent. Paroxysmal (or intermittent) AF involves episodes that cease within seven days with or without intervention. Most of these patients will have recurrent episodes. Previous literature has suggested that up to 90% of intermittent AF cases can recur. In addition, these episodes may have overt clinical manifestations or be silent. Furthermore, progression to a more sustained AF (persistent or permanent AF) is possible. Persistent AF involves episodes that do not terminate within seven days and require intervention to revert to sinus rhythm. Long-standing persistent AF may last more than one year. And permanent AF is persistent AF in which rhythm control therapy is no longer used. A patient can progress to any of these types of AF throughout their life [1].

When assessing a patient presenting with AF, it is vital to determine whether the arrhythmia is due to reversible causes such as hyperthyroidism or sepsis. These underlying issues should be addressed prior to initiating rate or rhythm control. Treatment of primary AF is based on the patient’s stability. Unstable patients require immediate electrical cardioversion and anticoagulation for four weeks after cardioversion [2]. The unstable patients include those with respiratory distress, poor perfusion, altered mental status, or shock.

Patients with rapid heart rates are at risk of cardiomyopathy and heart failure (due to loss of atrial kick) [3]. This can be controlled using an intravenous BB or CCB. These therapies slow conduction at the AV node, thus lowering the ventricular rate. These agents should not be utilized in patients with severe hypotension or in pre-excitation syndromes. Furthermore, because these agents block the AV node, there is a risk of heart blocks with their use. Digoxin or amiodarone can be used if BBs and CCBs are ineffective or contraindicated [1,2], but the former has too slow an onset to be used in the ED setting.

Previous studies have shown no difference in morbidity or mortality between therapy with rate or rhythm control modalities [4,5]. Converting to sinus rhythm from AF has been thought to decrease the risk of thrombotic events, but there have been issues raised with the adverse effects of antiarrhythmic drugs such as amiodarone, flecainide, propafenone, and others. In addition, there have been studies demonstrating increased hospitalizations with rhythm control [6,7]. Rate control modalities are known to be more cost-effective, which patients may opt for [8]. Rate control is preferred in patients with increased age due to the adverse effects of antiarrhythmic agents, reduced clearance in the elderly, and increased risk of proarrhythmias [9]. Rhythm control is preferred when rate control has failed or in patients who are younger (less than 65 years of age).

New-onset AF should be treated with anticoagulation due to increased risk of thrombotic events. In patients with pre-existing AF, the CHADS-VASc score can be utilized to decide whether a patient should be treated with anticoagulation with warfarin or direct oral anticoagulations (DOACs) [10]. This clinical prediction tool can stratify patients with non-rheumatic AF against their risk of a stroke. It incorporates data such as age, sex, history of congestive heart failure, hypertension, thrombotic event, vascular disease, and diabetes in calculating scores. A score of 0 indicates low risk, not requiring anticoagulation. A score of 1 is low-to-moderate risk and antiplatelet or anticoagulation should be considered. A score of 2 or more is moderate-to-high risk and anticoagulation is recommended. Patients with paroxysmal AF do have a lower 30-day risk of thrombotic event than those with sustained AF. However, duration and frequency in AF are important factors in deciding whether to begin anticoagulation. One study found that each additional hour of AF burden (including silent AF) increased the risk of stroke by about 3% [11]. Therefore, anticoagulation can be considered in patients with paroxysmal AF and lower prediction scores, like in our patient.

## Conclusions

This report described the case of a middle-aged man who presented with AF with RVR and went into sudden cardiac arrest following treatment with diltiazem. The patient had ROSC and eventually reverted back to sinus rhythm. This report serves as a reminder that no intervention is benign. When administering a drug to decrease RVR in AF, it is important to closely monitor vital signs and have resuscitative equipment at the ready.
